# Multifidus Fat Infiltration in Patients with Persistent Spinal Pain Syndrome Type II Treated with Spinal Cord Stimulation: A Preliminary Report

**DOI:** 10.3390/jcm14093124

**Published:** 2025-04-30

**Authors:** Maarten Moens, Laurène V. Genot, Frederick Van Gestel, Julie G. Pilitsis, Maxime Billot, Manuel Roulaud, Philippe Rigoard, Lisa Goudman

**Affiliations:** 1 STIMULUS Research Group, Vrije Universiteit Brussel, Laarbeeklaan 103, 1090 Brussels, Belgium; 2 Cluster Neurosciences, Center for Neurosciences (C4N), Vrije Universiteit Brussel, Laarbeeklaan 103, 1090 Brussels, Belgium; 3 Department of Neurosurgery, Universitair Ziekenhuis Brussel, Laarbeeklaan 101, 1090 Brussels, Belgium; 4 Pain in Motion (PAIN) Research Group, Department of Physiotherapy, Human Physiology and Anatomy, Faculty of Physical Education and Physiotherapy, Vrije Universiteit Brussel, Laarbeeklaan 103, 1090 Brussels, Belgium; 5 Department of Radiology, Universitair Ziekenhuis Brussel, Laarbeeklaan 101, 1090 Brussels, Belgium; 6 Research Foundation—Flanders (FWO), 1090 Brussels, Belgium; 7 NEUR, Center for Neurosciences (C4N), Vrije Universiteit Brussel, Laarbeeklaan 103, 1090 Brussels, Belgium; 8 Department of Neurosurgery, University of Arizona College of Medicine, 1501 N Campbell Ave, Tucson, AZ 85724, USA; 9 PRISMATICS Lab (Predictive Research in Spine/Neuromodulation Management and Thoracic Innovation/Cardiac Surgery), CHU de Poitiers, 86000 Poitiers, France; 10 Service de Neurochirurgie du Rachis, Chirurgie de la Douleur et du Handicap, CHU de Poitiers, 86000 Poitiers, France; 11 Pprime Institute UPR 3346, CNRS, ISAE-ENSMA, Université de Poitiers, 86000 Poitiers, France

**Keywords:** manual segmentation, neuromodulation, magnetic resonance imaging, paravertebral muscles, lumbar spine

## Abstract

**Background/Objectives**: Functional spinal instability from multifidus dysfunction has been proposed as a mechanism for chronic postsurgical pain. Prior studies reported structural impairments in the lumbar multifidus in patients with chronic low back pain, including a reduced cross-sectional area, muscle thickness, and increased fat infiltration. This preliminary report examined the prevalence of multifidus fat infiltration after Spinal Cord Stimulation (SCS), an established pain management technique. It also assessed inter-rater reliability in evaluating fat infiltration using MRI. **Methods**: The medical imaging data from four patients with Persistent Spinal Pain Syndrome Type II (PSPS II) treated with SCS were collected. Two independent operators performed the manual segmentation of the multifidus muscle on axial MRI images of the lumbar spine. The fat-to-muscle ratio was quantified and rated using a four-point classification system, categorizing multifidus fat infiltration as normal, mild, moderate, or severe. To assess the reliability of the manual segmentations, inter-rater reliability was determined. **Results**: The median fat-to-muscle ratio at the levels L2–L3 was 46.12 (Q1–Q3: 44.88–47.35). At the levels L3–L4, L4–L5, and L5–S1, the median values were 50.45 (Q1–Q3: 45.57–52.98), 52.11 (Q1–Q3: 48.81–52.80), and 52.84 (Q1–Q3: 49.09–56.39), respectively. An ICC value of one (95% CI from 0.999 to 1, *p* < 0.001) was found for inter-rater agreement on the muscle volume of the multifidus muscle. **Conclusions**: All the patients had moderate-to-severe fat infiltration of the multifidus muscle at each lumbar spinal level. Although time-consuming, the manual segmentation of the multifidus muscle in patients treated with SCS was feasible and yielded excellent inter-rater reliability when determining muscle volume. Future endeavors should focus on the automation of segmentation and classification.

## 1. Introduction

It remains unclear which patients will develop long-lasting pain after surgery [1], likely due to the multifactorial nature of chronic pain and the complex interaction between internal and environmental factors that contribute to its chronicity [2]. Functional spinal instability resulting from multifidus muscle dysfunction is often proposed as one of the underlying mechanisms for pain chronicity [3]. The multifidus muscle includes short fibers that cross as few as two segments (referred to as the deep multifidus), which are responsible for compression and segmental control with a limited extension moment. In contrast, progressively longer fibers cross up to five segments (referred to as the superficial multifidus), which are mainly responsible for spine extension or stiffness when cocontracting with antagonist muscles [4,5]. The multifidus muscle exhibits an architectural design (i.e., a large cross-sectional area and low fiber-length-to-muscle-length ratio) enabling unique stabilizing properties, allowing for it to produce large forces [6].

Structural impairments of the lumbar multifidus muscle, such as a reduced cross-sectional area [7,8], reduced thickness [9], and increased fat infiltration [10,11], have been documented in patients with chronic low back pain [12]. Furthermore, higher electromyography activity in the lumbar erector spinae and the multifidus muscle has been observed in patients with chronic low back pain compared to those of healthy controls, suggesting enhanced muscle activity [13]. A systematic review confirmed the negative association between the cross-sectional area of the multifidus muscle and low back pain [14]. When comparing patients with chronic low back pain with low versus high pain intensity scores (cut-off score of 6/10), the patients with low pain intensity scores did not differ from the controls without back pain, while the patients with high pain intensity scores had a 24.3% higher multifidus fat fraction [11]. In patients who underwent surgical treatment, an association between low multifidus fat infiltration at the baseline (as assessed by MRI) and greater improvement in low back pain was found [15].

Advances in medical imaging have diversified the methods used to assess lumbar muscle atrophy [16]. Initially, the cross-sectional area was measured via computed tomography or real-time ultrasound [16,17]. The development of high-resolution magnetic resonance imaging (MRI) has since made it the primary tool for assessing lumbar muscle atrophy through threshold-based segmentation to better distinguish muscle from fat [16,18,19].

When experiencing chronic low back pain, exercise therapy in the form of lumbar stabilization exercises, specifically targeting the multifidus muscle, has been shown to reduce low back pain intensity and lower the recurrence rate [12,20]. Additionally, targeting the control and degeneration of the multifidus muscle with restorative neurostimulation has been proposed as a new treatment approach for nociceptive chronic low back pain due to vertebral joint overload following the functional instability of the lumbar spine [21,22]. Instead of focusing on restorative neurostimulation for nociceptive pain, the current preliminary report evaluates the prevalence of multifidus fat infiltration after Spinal Cord Stimulation (SCS), a treatment known to reduce pain, improve functionality, and enhance quality of life in patients with neuropathic pain by activating the dorsal columns through an electrical current [23,24]. The Gate Control Theory initially explained the mechanism of SCS, proposing that the stimulation of large-diameter nerve fibers inhibits nociceptive signal transmission at the dorsal horn of the spinal cord [25,26]. In addition to segmental mechanisms, various supraspinal mechanisms have been proposed. The most frequently reported ones involve the modulation of the descending nociceptive inhibitory pathways, followed by changes in the ascending medial and lateral pathways, as evidenced by both human and animal studies [27]. To the best of our knowledge, the direct effect of SCS on multifidus fat infiltration has not yet been elucidated. However, the literature already demonstrated increases in physical activity of 40% or more at every time point when pain was reduced by SCS [28]. Additionally, physical therapy in patients with chronic low back pain has been shown to reduce fat infiltration at the lumbar level [29,30]. Based on these findings, the authors propose that SCS improves physical activity, and consequently may alter muscle composition by reducing fat infiltration. Before the introduction of MRI compatibility in implanted neurostimulation devices, there were significant barriers preventing patients with implanted SCS systems from undergoing MRI scans. However, subsequent modifications to the design of both the leads and the implantable pulse generator altered the dissipation of energy absorbed by tissues to comply with the FDA-specific absorption rate requirements, leading to the development of MRI-conditional devices [31]. It remains unclear whether multifidus fat infiltration persists in patients who have undergone SCS, and subsequently experience reduced pain and improved functionality [32]. Therefore, the primary aim of this study is to evaluate the prevalence of multifidus fat infiltration after SCS, as determined on MRI images. The secondary aim of this preliminary report is to assess inter-rater agreement in measuring fat infiltration from MRI images using a manual segmentation approach.

## 2. Materials and Methods

### 2.1. Study Protocol

This retrospective study was conducted using the data gathered in a clinical practice from patients with Persistent Spinal Pain Syndrome Type II (PSPS-T2) at the department of Neurosurgery, UZ Brussel, as well as MRI images of these patients. The patients first underwent the implantation of the Intellis^TM^ implantable pulse generator (Medtronic, Minneapolis, MN, USA) followed by an MRI scan of the lumbar spine performed at least 6 months after implantation to assess the multifidus muscle.

The study protocol of this retrospective study was approved by the ethics committee of UZ Brussel on 18 January 2023 (B.U.N. 1432022000260).

### 2.2. Participants

The data from 1 January 2018 to 18 November 2022 were collected from patients (aged ≥ 18 years) diagnosed with PSPS-T2 and treated with the MRI-compatible SCS-type Intellis^TM^. These patients presented at the outpatient clinic of the Department of Neurosurgery, UZ Brussel, for their 6-month follow-up care. PSPS-T2 is defined as persistent neuropathic pain of radicular origin with pain in the lower back and/or leg(s), with an intensity of at least 4/10 on the numeric rating scale, lasting for a minimum of six months, following at least one anatomically successful spinal surgery and refractory to conservative treatment (according to the Belgian reimbursement rules from 1 January 2018). The patients were included if lumbar spine MR images from L1 to L5 were available, acquired at least six months post-implantation. The exclusion criteria included conditions known to cause muscle wasting (e.g., multiple sclerosis, muscular dystrophy, amyloid lateral sclerosis, and malnutrition) or poor quality MR images. The demographic data were extracted from each patient, including sex, age (in years), height, and weight.

### 2.3. Segmentation Composition Analysis

**Figure 1:**
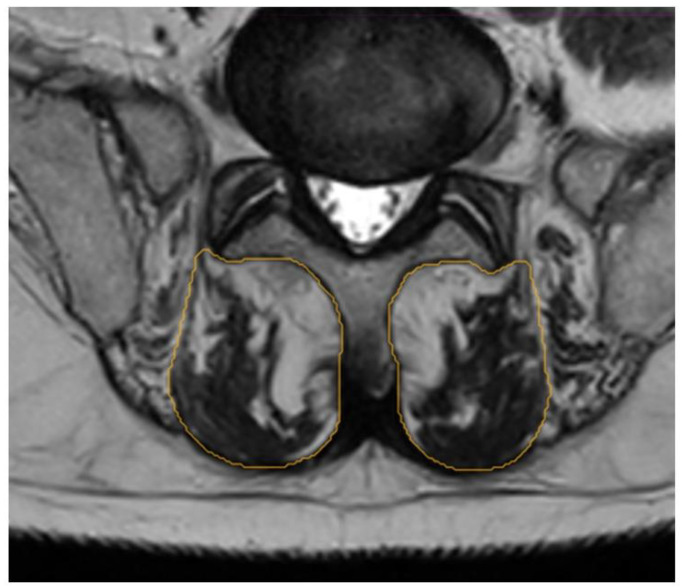
Example of region of interest for manual segmentation of multifidus muscle at level L5–S1.

The segmentation of the multifidus muscle (ROI) was performed manually on the axial slices of both T1- and T2-weighted MRI at the lumbar level (L2–L3, L3–L4, L4–L5, and L5–S1). For image acquisition, the following parameters were used: field strength (from 1.5 to 3 Tesla), Echo Time (from 13 to 120 ms), and slice thickness (3.0 mm). Coronal and sagittal images were also used, along with CT scans if available, to perform image fusion between MRI and CT to enhance accuracy in outlining the ROI and assessing intra-rater reliability. A single independent operator performed all manual segmentations using a standardized procedure for identifying the multifidus muscle on axial slices as described by Crawford et al. [33]. In this approach, the borders of the muscles were defined as follows: the medial border by the most superficial aspect of the spinous processes, the anterior margin by the superficial edge of the lamina of the vertebral body to the mamillary process, the lateral border as the epimysium of the spino-transverse muscle group, and the posterior demarcation includes the superficial aspect of the epimysium that separates the thoracolumbar fascia from the multifidus muscle. Certain modifications were made for this study, which specifically focused on evaluating fat infiltration. In this context, the middle of the fat deposits between the multifidus and erector spinae muscles was considered the lateral margin. Muscle tendons were excluded from segmentation to avoid potential bias in quantifying fat infiltration. An example of this segmentation is presented in Figure 1.

**Figure 2:**
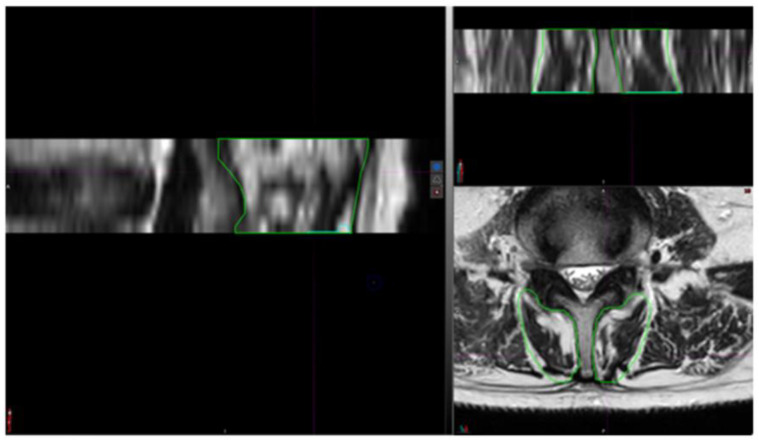
Segmentation of multifidus muscle through axial, coronal, and sagittal axes.

To assess the reliability of the manual segmentations, both intra- and inter-rater reliability were evaluated. Intra-rater reliability was established by cross-checking the segmentation of the axial sections with reference to the coronal and sagittal views, as well as through MRI/CT fusion (Figure 2). This process allowed for the operator to verify the accuracy of the segmentations and confirm the absence of abnormalities.

**Figure 3:**
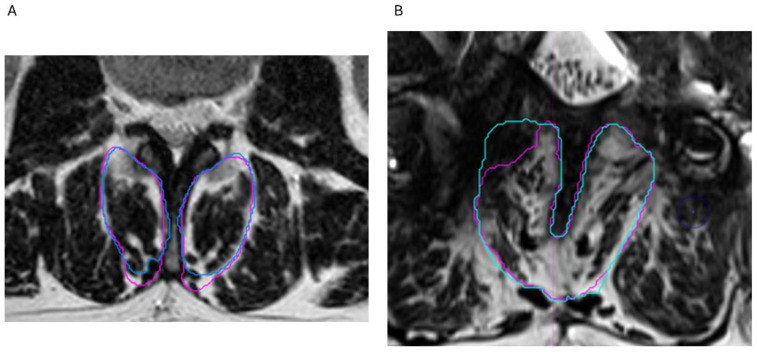
Manual segmentation by 2 operators. (**A**) Segmentation by operator 1 (blue) and operator 2 (pink). (**B**) Scar tissue was not included by operator 2.

Inter-rater reliability was determined by having a second operator review, and if necessary, adjust all the segmented images created by the first operator (Figure 3). Reliability was quantified by comparing the muscle volumes derived from both the sets of segmentations. Finally, all the segmentations at each level were reconstructed into volumes to determine muscle volume per lumbar level.

The fat infiltration of the multifidus muscle (region of interest) was assessed by measuring the replacement of muscle with adipose tissue on the segmented axial MRI images of the lumbar spine (L2–L3, L3–L4, L4–L5, and L5–S1). A multi-segment approach was used, as it provides a more accurate evaluation of overall fat infiltration than a single-level assessment [34]. Each segmented level was reconstructed into a volume, and a threshold algorithm was applied to distinguish muscle from fat based on signal intensity. Due to the variability in grey scale and contrast weighting across the patients’ MR images, the threshold value was individually set based on visual assessment, ranging from 390 to 500.

Once the threshold was defined, muscle and fat volumes were calculated. The fat-to-muscle ratio was then used to determine the average fat infiltration at each lumbar level. The results were classified using a four-grade scale: grade 1 (<10%) indicated normal fat infiltration; grade 2 (10–30%) was mild; grade 3 (30–50%) was moderate; and grade 4 (>50%) was severe [35]. In addition to this objective analysis, the visual estimation of fat infiltration per level was also performed using the same four-grade classification (referred to as visual grading).

### 2.4. Statistical Analysis

Statistical analyses were performed using RStudio version 2022.07.2. The descriptive statistics are provided as absolute numbers of observations with corresponding percentages. In all analyses, *p*-values of 0.05 or less were considered statistically significant.

Inter-rater agreement between the measured muscle volumes of both the operators was assessed using intraclass correlation (two-way model and single rating) and Cohen’s Weighted Kappa for the degree of fat infiltration of the visually graded images. To evaluate the consistency of the visual grading, the Kendall Rank correlation coefficient was determined for the visually and quantitatively measured fat content classification. Lastly, the correlation between the lumbar vertebral level and fat infiltration was analyzed using the rmcorr package in RStudio, which provides a measure of repeated measure correlation.

## 3. Results

### 3.1. Demographics

Out of 27 patients, 10 patients had available MRI images. Of these 10 patients, the MRI images were taken before implantation in six patients. Therefore, the data of four patients (one male and three females) was used in this preliminary report. The participant demographics are summarized in Table 1. The patients had a median age of 47 years (Q1–Q3: 46.5–50.5 years) at the time of the lumbar MRI scan. The median time between implantation and the MRI scan entailed 991 days (Q1–Q3: 338–1606 days). The median BMI was 28.45 kg/m^2^ (Q1–Q3: 26.95–32.42 kg/m^2^). One patient had a previous surgery at L2–L3, while the remaining three patients had surgery at L4-L5, and one patient also had surgery at L5–S1 in addition to L4–L5. For two patients, no data were available at level L1–L2 due to the lack of full spine MRI images.

### 3.2. Prevalence of Fat Infiltration

**Figure 4:**
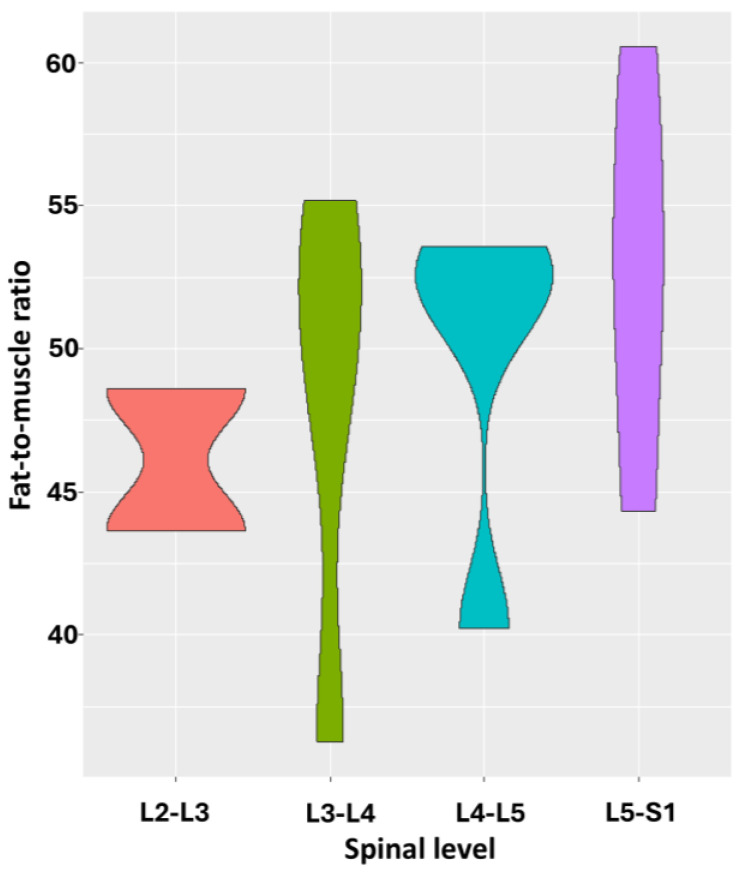
This violin plot shows relation between spinal level and fat-to-muscle ratio. Wider sections of violin plot represent higher probability that members of population will take on given value; skinnier sections represent lower probability.

The median fat-to-muscle ratio at the levels L2-L3 was 46.12 (Q1–Q3: 44.88–47.35), revealing grade 3 fat infiltration for both the patients. At the levels L3–L4, L4–L5, and L5–S1, the median values of 50.45 (Q1–Q3: 45.57–52.98), 52.11 (Q1–Q3: 48.81–52.80), and 52.84 (Q1–Q3: 49.09–56.39) were found, respectively (Figure 4). Translating these ratios into a classification system for multifidus fat infiltration at the levels L3–L4, two patients demonstrated grade 3 fat infiltration, and two patients had grade 4 infiltration. At the levels L4–L5 and L5–S1, one patient revealed grade 3 infiltration, and three patients had grade 4 fat infiltration.

### 3.3. Reliability Analysis

The inter-rater reliability results for the manually segmented muscles and the visual grading of the degree of fat infiltration are presented in Table 2. In absolute numbers, the differences in muscle volume between both the operators was 1.6 for a total volume of 569.63 mL, leading to overall agreement of 99.72%. The median muscle volume of 42.51 mL (Q1–Q3: 29.32–49.67 mL) was measured by both the operators, without significant difference (V = 4, *p* = 0.79). An ICC value of 1 (95% CI from 0.999 to 1, *p* < 0.001) was found for inter-rater agreement on the muscle volume of the multifidus muscle.

In addition, the inter-rater agreement for the visual grading of the degree of muscle fat infiltration showed good results overall (kappa = 0.759, *p* < 0.001). More specifically, poor absolute agreement between the two operators was found for L3–L4 (kappa = 0.5, *p* = 0.18), while excellent agreement was revealed at the levels L4–L5 and L5–S1.

Comparing the degrees of classification derived from manual segmentation versus visual grading, an overall moderate positive correlation was revealed at all the levels (r = 0.56, *p* = 0.04).

### 3.4. Fat Infiltration over Lumbar Levels

**Figure 5:**
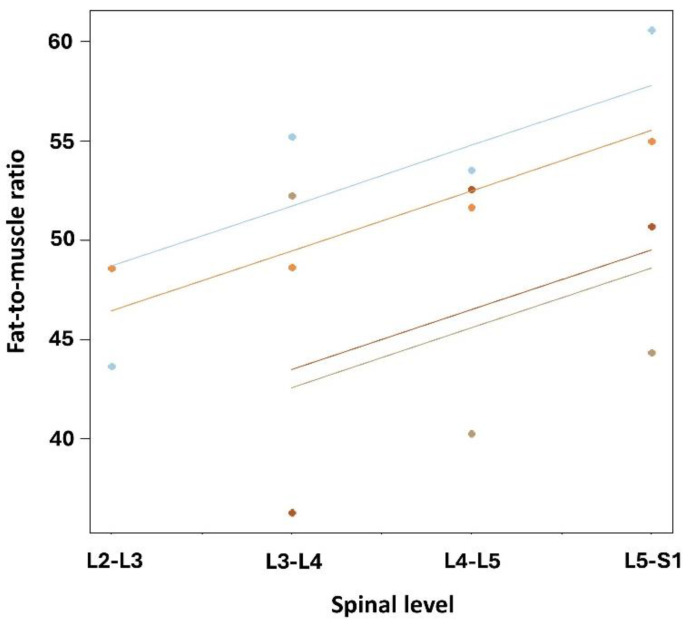
Repeated measure correlation between fat infiltration and lumbar spine level for all patients. At intra-individual level (i.e., repeated spine levels), non-significant positive correlation was observed between higher degrees of fat infiltration and lower spinal levels (r = 0.56, *p* = 0.07). Observations shown in same color corresponded to same patient.

Repeated measure correlation revealed a nonsignificant positive relationship (r = 0.56, *p* = 0.07) between more fat infiltration and the lower lumbar levels at the individual level (Figure 5).

## 4. Discussion

This study demonstrated the feasibility of evaluating fat infiltration on axial MRI images using manual segmentation in patients who are treated with SCS, enabled by advances in MRI-compatible SCS technology. As the need for MRI has become more common for diagnosis and disease surveillance, SCS device selection based on MRI compatibility has gained a lot of importance [36]. From approximately 82% to 84% of SCS-implanted patients are expected to need at least one MRI within five years after implantation [37]. Furthermore, from 59% to 74% of patients will require a non-spinal MRI within ten years after implantation [37].

The potential risks of MRI with SCS include transient alterations in pulse rate, amplitude, pulse width, and electrode configuration [36]. Specifically for the implantable pulse generator, transient warmth sensations around the pocket, battery exhaustion, and the (de)activation of the pulse generator are among the potential risks [36]. The Intellis^TM^ pulse generator, combined with Medtronic Vectris^TM^ leads or Specify^®^ Surescan^®^ MRI leads (Medtronic, Minneapolis, USA), is MRI-conditional under on-label implantation conditions nowadays [38]. The term “MRI-conditional” refers to devices that are safe in specific MRI environments if the conditions related to the scanner, the device, and the patient are met [39]. These technological advancements indicate that providing SCS to patients no longer jeopardizes future healthcare for patients with chronic pain. Prior to implantation, counselling for patients regarding the use of MRI-conditional SCS systems should be carefully considered [40,41].

When assessing muscle composition, comparisons between studies are often challenging due to the differences in segmentation (e.g., manual, semi-automated, or automated segmentation tools), the broad variety in software, and the different methods used to define the regions of interest for each muscle [33]. In this study, we focused on axial MRI images to conduct the manual segmentation of the multifidus muscle.

Before relying on this manual approach, a semi-automatic approach was assessed using MIM software (version 7.1.2, MIM Software, Cleveland, OH, USA) as an artificial intelligence learning system that can semi-automatically define a particular region of interest after training with a dataset of processed images [42]. During the training process, several problems occurred (e.g., rotating MRI images due to a lack of continuous axial cuts, and the lateral cropping of sagittal images, causing a lack of information about lateral aspect of muscles), which made a semi-automatic approach unfeasible, so a manual approach was adopted. This highlights the necessity for standardized MRI protocols to ensure comprehensive and complete axial lumbar MR scans. When evaluating the inter-rater reliability with this manual approach, excellent agreement was found when determining muscle volume, a necessary step in calculating the fat-to-muscle ratio, which points towards the robustness of this approach.

A pure visual evaluation resulted in a substantial agreement between both the operators, with a significant moderate correlation between the degree of fat infiltration based on manual segmentation versus visual grading. These results point towards the strength of manual segmentation techniques, whereby visual grading classifications could have a value in urgent situations, yet preferably not in daily clinical routine situations.

All the patients in this study exhibited moderate-to-severe fat infiltration in the multifidus muscle following SCS implantation. No clear pattern of increased infiltration at the lower lumbar levels was observed, likely due to the small sample size and limited statistical power. Prior research has linked a better multifidus muscle quality to lower levels of back-related disability and leg pain, suggesting its relevance in lumbar spine disorders.

Despite treatment with SCS, the patients with PSPS-T2 continued to show significant fat infiltration, highlighting the need for targeted interventions. Currently, the guidelines for rehabilitation after spinal surgery, particularly for those with persistent or recurrent pain, remain limited [43]. Post-SCS physical therapy emphasizes individualized care, considering patients’ needs and risks, such as lead migration or hardware damage [44].

Based on these findings, lumbar stabilization and motor control exercises targeting the multifidus muscle [20] should be more prominently integrated into post-SCS rehabilitation. Restorative neurostimulation may also benefit patients with mechanical pain due to multifidus dysfunction, especially when surgery is not indicated [45]. This therapy was developed to electrically stimulate the medial branch of the L2 dorsal ramus nerve to elicit isolated multifidus muscle activation [46]. This approach activates the motor neurons to restore multifidus function and segmental stability [47]. A recent five-year cohort study supports its long-term benefits in pain reduction, disability, and quality of life, with a favorable safety profile [48].

Given these findings, assessing multifidus condition prior to SCS implantation could help guide treatment decisions, either toward rehabilitation or restorative neurostimulation. Fat infiltration may serve as a valuable stratifying variable in personalized chronic pain management, offering a more objective basis for therapy selection. Finally, both self-reported and objective measures are recommended for follow-up assessments in chronic pain settings [49], with fat infiltration potentially serving this role.

This study has several limitations that should be considered when interpreting the results, and which affect the generalizability of the findings. First, this study is a preliminary report in which only 14 lumbar levels were evaluated from four patients. This study could have benefitted from a larger sample size for sufficient power to answer the research question, wherefore this report merely serves as a starting point to provide pilot data to substantiate sample size calculations for larger trials in this population.

Second, the cross-sectional study design precludes the ability to conduct the longitudinal follow-up of the MRI images of these patients. Future studies could address this limitation by investigating whether fat infiltration in patients with PSPS Type II can be reduced over time and whether specific treatments lead to greater reductions. These studies can conduct a proper sample size calculation based on the data provided in this preliminary report to detect meaningful differences in multifidus fat infiltration. The lack of longitudinal data also limited causal inference, making it unclear whether fat infiltration was caused by SCS, underlying pathology, or normal aging. To address this, a longitudinal trial should have been conducted in the patients with PSPS T2 who were randomly assigned to receive either SCS or usual care. A control group of healthy participants, matched for gender and age with the patients, should also have been included and subjected to the same follow-up measurements. This design would have allowed for causal inferences.

Third, the manual segmentation and variations in the MRI gray matter values between the patients may have introduced limitations to the accuracy of the measurements. Finally, this study focused exclusively on multifidus fat infiltration at the lumbar levels and did not assess multifidus fat infiltration at the cervical levels, despite a standardized neck pain prevalence rate of 27.0 per 1000 individuals in 2019 [50]. This could be conducted in future research since patients with PSPS T2 could experience lumbar pain as well as cervical pain, and for both these locations exercise therapy and neurostimulation are potential treatments [51,52].

## 5. Conclusions

The manual segmentation of axial MRI images from patients with PSPS T2 who are treated with SCS is a feasible technique with excellent inter-rater reliability for determining multifidus muscle volume. The visual grading classifications revealed substantial inter-rater agreement between both the operators, with moderate correlations between the degree of fat infiltration based on segmentation versus visual grading. This may limit the value of visual grading in daily clinical routine. This preliminary report pointed towards the moderate-to-severe fat infiltration of the multifidus muscle in all the patients at each lumbar spinal level.

## Figures and Tables

**Table 1:** Patient demographics.

Patient Profile	BMI (kg/m^2^)	Level of Surgery	IPG Implantation	Pain Medication	Pain Duration
Male, 61 y	26.2	L2–L3	2022	Lyrica	11 years, after herniectomy L2–L3
Female, 47 y	29.7	L4–L5	2021	Tramadol retard	5 years, after lumbar fusion L4–L5
Female, 45 y	40.6	L4–L5, L5–S1	2018	Oxycodone, Lyrica, Diclofenac, Cymbalta	18 years, after disc herniation L5–S1 and 6 years later L4–L5
Female, 47 y	27.2	L4–L5	2018	Oxycontin, Oxynorm, NSAIDs	6 years, after L4–L5 ALIF

Abbreviations—IPG: implantable pulse generator; y: years.

**Table 2:** Inter-rater reliability analysis of muscle volume and classification for multifidus fat infiltration.

Level	Contingency Table				Intraclass Correlation Coefficient [95% CI] for Muscle Volume	Cohen’s Weighted Kappa [95% CI] for Visual Grading	Kendall’s tau Correlation for Segmentation Versus Visual Grading
**L2–L3 (n = 2)**		**Grade 2 (R2)**		**Grade 3 (R2)**	0.969 [0.248 to 1], *p* = 0.01	NA	NA
	**Grade 2 (R1)**	0		1			
	**Grade 3 (R1)**	0		1			
**L3–L4 (n = 4)**		**Grade 2 (R2)**		**Grade 3 (R2)**	1 [1 to 1], *p* < 0.001	0.5 [−0.235 to 1], *p* = 0.18	r = 0, *p* = 1
	**Grade 2 (R1)**	2		0			
	**Grade 3 (R1)**	1		1			
**L4–L5 (n = 4)**		**Grade 2 (R2)**		**Grade 3 (R2)**	1 [1 to 1], *p* = 1	1 [1 to 1]	r = 1, *p* = 0.08
	**Grade 2 (R1)**	1		0			
	**Grade 3 (R1)**	0		3			
**L5–S1 (n = 4)**		**Grade 2 (R2)**	**Grade 3 (R2)**	**Grade 4 (R2)**	0.999 [0.994 to 1], *p* < 0.001	1 [1 to 1]	r = 0.77, *p* = 0.16
	**Grade 2 (R1)**	1	0	0			
	**Grade 3 (R1)**	0	2	0			
	**Grade 4 (R1)**	0	0	1			
**All levels (n = 14)**		**Grade 2 (R2)**	**Grade 3 (R2)**	**Grade 4 (R2)**	1 [0.999 to 1], *p* < 0.001	0.759 [0.430 to 1], *p* < 0.001	r = 0.56, *p* = 0.04
	**Grade 2 (R1)**	4	1	0			
	**Grade 3 (R1)**	1	7	0			
	**Grade 4 (R1)**	0	0	1			

Abbreviations—CI: confidence interval; n number of; NA: not applicable; R: operator.

## Data Availability

The data can be requested from the corresponding author based on reasonable request.
